# A Strategy for Discovery and Verification of Candidate Biomarkers in Cerebrospinal Fluid of Preclinical Alzheimer’s Disease

**DOI:** 10.3389/fnmol.2018.00483

**Published:** 2019-01-07

**Authors:** Xiaofang Zhong, Jingxin Wang, Cynthia Carlsson, Ozioma Okonkwo, Henrik Zetterberg, Lingjun Li

**Affiliations:** ^1^School of Pharmacy, University of Wisconsin-Madison, Madison, WI, United States; ^2^Neuroscience Training Program, University of Wisconsin-Madison, Madison, WI, United States; ^3^School of Medicine and Public Health, University of Wisconsin-Madison, Madison, WI, United States; ^4^Institute of Neuroscience and Physiology, Sahlgrenska Academy, University of Gothenburg, Gothenburg, Sweden; ^5^Clinical Neurochemistry Laboratory, Sahlgrenska University Hospital, Mölndal, Sweden; ^6^Department of Neurodegenerative Disease, UCL Institute of Neurology, London, United Kingdom; ^7^UK Dementia Research Institute at UCL, London, United Kingdom; ^8^Department of Chemistry, University of Wisconsin-Madison, Madison, WI, United States

**Keywords:** cerebrospinal fluid, Alzheimer’s disease, biomarker, label-free quantitation, targeted quantitative proteomics, isotopic labeling for quantitation, iDiLeu

## Abstract

Alzheimer’s disease (AD), a progressive neurodegenerative disease, is characterized by the accumulation of senile plaques, neurofibrillary tangles, and loss of synapses and neurons in the brain. The pathophysiological process of AD begins with a long asymptomatic phase, which provides a potential opportunity for early therapeutic intervention. Therefore, it is crucial to define putative biomarkers via reliable and validated methods for early diagnosis of AD. Here, we characterized candidate biomarkers by discovery proteomics analysis of cerebrospinal fluid (CSF), revealing that 732 and 704 proteins with more than one unique peptide were identified in healthy controls and preclinical AD patients, respectively. Among them, 79 and 98 proteins were significantly altered in preclinical AD for women and men, respectively, many of which have been demonstrated with consistent regulation pattern in patients with mild cognitive impairment or AD dementia. In-house developed 5-plex isotopic *N,N*-dimethyl leucine (iDiLeu) tags were further utilized to verify candidate biomarkers, neurosecretory protein VGF (VGF) and apolipoprotein E (apoE). By labeling peptide standards with different iDiLeu tags, a four-point internal calibration curve was constructed to allow for determination of the absolute amount of target analytes in CSF through a single liquid chromatography-mass spectrometry run.

## Introduction

Alzheimer’s disease (AD) is the most common form of dementia among the elderly. It is characterized by deposition of peptide amyloid-β (Aβ) as amyloid plaques and of protein tau as neurofibrillary tangles (Blennow et al., 2006). Complemented with molecular imaging techniques, cerebrospinal fluid (CSF) markers have been established to reflect AD pathologies (Blennow et al., 2010; Zetterberg, 2015). Emerging clinical cohort studies suggest that subtle cognitive alteration begins years before mild cognitive impairment (Sperling et al., 2011). It is crucial to establish a panel of reliable protein biomarkers for diagnosis of AD at very earliest stage (Hampel et al., 2010). CSF Aβ42 is a well-established marker of Aβ plaque pathology that is highly predictive of AD in cognitively normal individuals (Dubois et al., 2016). Although several peptide and protein biomarkers in CSF have been used for aiding in AD diagnosis (Puchades et al., 2003; Hall et al., 2012), an unambiguous diagnosis of AD at preclinical stage is still lacking. To establish novel and reliable biomarkers capable of early diagnosis in preclinical AD, mass spectrometry (MS) is implemented for unbiased candidate protein biomarker discovery as the principal enabling technology. MS-based label-free quantification holds special promise to discover significant disease-related protein dysregulations for large cohorts with high throughput and sensitivity. The potential biomarkers can be further verified through targeted proteomics with high accuracy and reproducibility (Peterson et al., 2012).

Enzyme-linked immunosorbent assay (ELISA) is widely used for biomarker verification in clinical laboratories, but the dependence on high-quality antibodies restrict its feasibility to quantify novel protein biomarkers (Hüttenhain et al., 2009). Absolute quantification (AQUA) strategy was first proposed to use known concentrations of synthesized heavy isotope-encoded peptides as internal standards to quantify concentrations of native peptides in the clinical sample (Gerber et al., 2003). However, the number of biomarker candidates to be verified would be limited due to the high cost of AQUA. Alternatively, mass difference labeling strategies such as stable isotope labeling by amino acids in cell culture (SILAC) (Ong et al., 2002; Seyfried et al., 2010), combined precursor isotopic labeling and isobaric tagging (cPILOT) (Robinson and Evans, 2012), amino acid-coded tagging (Pan et al., 2003), and mass differential tags for relative and absolute quantification (mTRAQ) (Kang et al., 2010) introduce heavy isotopes metabolically or chemically into target peptides to yield different precursor ion masses, which can be quantified through full MS scans. For example, mTRAQ can react with free N-terminus of a peptide through *N*-hydroxysuccinimide (NHS) ester group, there is no need to preselect target peptide to incorporate stable isotopes (Ross et al., 2004). mTRAQ is a relatively cost-efficient approach compared to AQUA, but like AQUA (Pannee et al., 2013), the three channels used for mTRAQ limit its ability to construct a standard curve with at most two channels to label standards and the third to label sample, leading to inaccurate estimates when protein amounts span a wide dynamic range in CSF.

**FIGURE 1:**
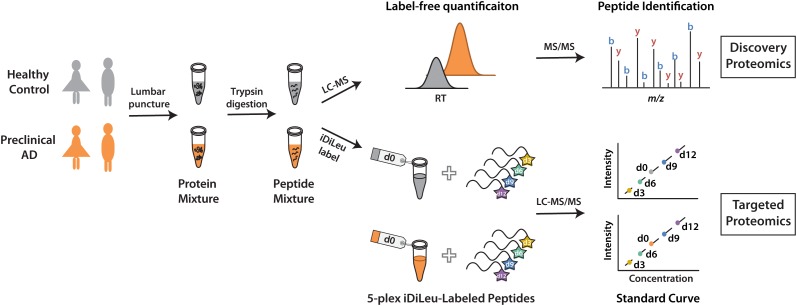
Workflow for label-free quantification-based discovery proteomics and isotopic labeling-based targeted proteomics.

The development of a set of five mass-difference reagents, isotopic *N,N*-dimethyl leucine (iDiLeu) tags, offers greatly reduced cost and significantly improves quantification throughput as compared to AQUA and mTRAQ (Greer et al., 2015). iDiLeu labeling strategy enables construction of a four-point calibration curve to quantify analytes in a single liquid chromatography–mass spectrometry (LC–MS) run. Each of the 5-plex iDiLeu reagents is comprised of an isotopic *N,N*-dimethyl leucine and an amine-reactive triazine ester moiety, which selectively labels the N-terminus of peptides as well as lysine side chains (Supplementary Figure S1). Mass additions of 141.1154, 144.1313, 147.1409, 150.1631, and 153.1644 Da are efficiently incorporated into peptides by d0, d3, d6, d9, and d12 labels, respectively. Different mass shifts can be discrete in the MS^1^ precursor ion scan for quantification, while tandem mass (MS^2^) yields abundant sequence-specific fragment ions for identification. Four iDiLeu labels are used to set up internal calibration curve by labeling peptide standards with known concentrations and the fifth channel is used to label target peptide of interest in CSF, making absolute quantification achievable based on the intensity of precursor ions in one LC–MS run. Here, we report a comparative global proteomics study in CSF from healthy and preclinical AD individuals to discover candidate biomarkers, which are further validated by targeted quantitative analysis with 5-plex iDiLeu tagging strategy (Figure 1).

## Materials and Methods

### Participants

Twelve enrollees (six individuals in preclinical AD stage and equal number of healthy controls) from Wisconsin Alzheimer’s Disease Research Center (ADRC) participated in this study. The participants comprised of three women in preclinical AD and three gender, family history, and years of education matched healthy controls respectively, as well as three paired men. Table 1 summarizes the demographic characteristics of the participants. Classification of participants as cognitively normal was based on a comprehensive neuropsychological test battery (Almeida et al., 2015). Global cognitive function was evaluated by Mini-Mental State Examination (MMSE), on which a score of ≤24 is deemed an indication of cognition impairment (Folstein et al., 1975). All participants had MMSE scores of ≥28, indicating cognition normal. Classification of preclinical AD was based on evidence of amyloid beta (Aβ) accumulation on ^11^C Pittsburgh Compound B Positron emission tomography (PET) imaging and hypometabolism on ^18^F Fluoro-2-deoxy-glucose (FDG)-PET. The University of Wisconsin Institutional Review Board approved all study procedures. All participants provided signed informed consent.

**Table 1:** Characteristics of the current study participants.

Diagnosis	Gender	Age (years)	Education (years)	MMSE score	Amyloid^∗^ (^11^C PiB-PET)	Glucose^∗^ (FDG-PET)	Family history of dementia (n)	ApoE 𝜀4 carriers (n)
Control *n* = 3	W	54.4 ± 3.8	15.3 ± 0.9	29.3 ± 0.5	-0.7 ± 0.3	0.5 ± 0.2	2	0
Predinical AD *n* = 3	W	58.4 ± 3.5	17.3 ± 1.2	29.0 ± 0.8	1.0 ± 11.3	-0.9 ± 0.5	3	3
Control *n* = 3	M	62.3 ± 2.5	15.3 ± 2.5	30.0 ± 0	-0.6 ± 0.3	1.2 ± 0.5	2	1
Predinical AD *n* = 3	M	63.8 ± 2.0	17.0 ± 0.8	29.7 ± 0.5	1.5 ± 1.6	-1.1 ± 0.8	2	1

*Data are presented as mean ± standard deviation. AD, Alzheimer’s disease; W, women; M, men; MMSE, Mini-Mental State Examination; ^11^C-PiB, ^11^C-labeled Pittsburgh compound B; PET, Positron emission tomography; FDG, ^18^F-fluorodeoxyglucose. ^∗^These measures are in arbitrary units (a.u.). The amyloid burden data (PiB-PET) were residualized for the effects of age and gender. The glucose metabolism data (FDG-PET) were residualized for the effects of age, gender, and global glucose metabolism.*

### CSF Protein Digestion

One mL CSF samples were collected through lumbar puncture. After 3 kDa molecular weight cut off separation, the concentrated protein was subjected to Sigma LC2 IgY column and 14 most abundant proteins were depleted to reduce sample complexity. 95–99% depletion efficiency was achieved and the unbound protein was measured by 660 nm protein assay kit (Thermo Scientific Pierce) at absorbance of 660 nm in Tecan Ultra 384 multi-detection microplate reader (Männedorf, Switzerland). Equal amounts of CSF proteins were reduced by adding dithiothreitol to a final concentration of 5 mM and incubated at room temperature for 1 h. 15 mM of iodoacetamide was used for alkylation of cysteines by incubating for 30 min at room temperature in the dark. After quenching unreacted iodoacetamide with 5 mM dithiothreitol, the protein mixture was diluted with 50 mM Tris buffer (pH 8) to a final urea concentration of 1 M prior to digestion with trypsin at a protein:enzyme ratio of 50:1 at 37°C for 16 h. The digestion reaction was quenched by acidification with 10% trifluoroacetic acid (TFA) to pH 3, followed by desalting with Bond Elut OMIX C18 pipette tips (Agilent Technologies, Santa Clara, CA, United States). For targeted proteomics, another one mL CSF samples were dried down in vacuum via centrifugation with a SpeedVac concentrator (Thermo Scientific, Waltham, MA, United States) and resuspended in 100 μL of lysis buffer, which contained 8 M urea, 50 mM tris base (adjust pH to 8 with hydrochloric acid), 5 mM CaCl_2_, 20 mM NaCl, and 1 tablet of EDTA-free protease inhibitor cocktail. CSF proteins were quantified and digested as above.

### Synthesis and Activation of iDiLeu Reagents

Synthesis of 5-plex iDiLeu isobaric tags was previously described in detail (Xiang et al., 2010; Greer et al., 2015). iDiLeu tags stored at -20°C are very stable until activation for labeling. The activation buffer consisted of 4-(4,6-dimethoxy-1,3,5-triazin-2-yl)-4-methylmorpholinium tetrafluoroborate (DMTMM) and *N*-methylmorpholine (NMM) at 0.6x molar ratios of iDiLeu tags dissolved in anhydrous *N,N*-dimethylformamide (DMF). Activation of iDiLeu tags was conducted by vortexing at room temperature for 1 h. The mixture was centrifuged at 14,000 ×*g* for 1 min and the supernatant was transferred immediately for peptide labeling.

### Peptides Labeling by 5-Plex iDiLeu Tags

A stock solution of synthesized neurosecretory VGF peptide (THLGEALAPLSK) was prepared to be 1 μg/μL. Five aliquots were set up for labeling with d0, d3, d6, d9, d12 iDiLeu reagent, respectively. In each aliquot, 20 μg of peptide standards were resuspended in 20 μL of 0.5 M triethylammonium bicarbonate (TEAB) buffer. To ensure sufficient labeling, peptide standards ware labeled with a 20x w/w excess of iDiLeu reagents. 60 μL of activated iDiLeu reagents were added to each peptide standards aliquot to make the organic:aqueous ratio of 75%. The reaction was vortexing for 2 h under room temperature. 50% hydroxylamine was added to final 0.25% to quench the reaction and incubated for another 10 min. Labeled peptides were vacuum-dried. Two hundred μg of apoE protein standard was digested based on the digestion procedure described above. Each of five aliquots containing 20 μg of apoE protein standard digests was labeled with the five-channel iDiLeu reagents individually.

For correction factor, 2.5 μg of peptide standards were reconstituted in strong cation exchange (SCX) reconstitution buffer. By-products of iDiLeu labeling reaction were removed from SCX SpinTips (Protea Biosciences, Morgantown, WV, United States) according to the manufacturer’s protocol. The eluate was dried in vacuo and desalted with ZipTip C18 pipette tips (Merck Millipore, Darmstadt, Germany).

Cerebrospinal fluid protein digests were reconstituted in 20 μL of 0.5 M TEAB and labeled with d0 reagent separately according to the peptide standards labeling procedure described above. The other four channels labeled peptide standards were spiked into each d0-labeled CSF sample in a ratio of 1:10:50:100. The combined sample was cleaned up with SCX SpinTips and desalted with Bond Elut OMIX C18 pipette tips. All the labeled samples were then dried in vacuo and reconstituted in 25 μL of 3% acetonitrile (ACN), 0.1% formic acid (FA) in water.

### LC–MS/MS Acquisition

Samples were analyzed using a Waters nanoAcquity ultra-performance liquid chromatography (UPLC) system (Milford, MA, United States) coupled to a Thermo Scientific Q-Exactive Orbitrap mass spectrometer (San Jose, CA, United States). Peptide mixture was loaded onto a fabricated column with an integrated emitter tip, which packed with Bridged Ethylene Hybrid C18 particles (75 μm × 150 mm, 1.7 μm, 130 Å). Mobile phase A was 0.1% FA in H_2_O and mobile phase B was 0.1% FA in ACN. Peptides were separated with a gradient elution of 4 to 35% B over 90 min at a flow rate of 300 nL/min. Full MS scan was acquired in profile mode ranging from *m/z* 350 to 1800 at a resolution of 70 K. Automatic gain control (AGC) target was 1 × 10^6^, and maximum injection time was 120 ms. Tandem mass spectra were acquired at centroid mode. The top 15 most abundant precursor ions were selected for higher-energy collisional dissociation (HCD) fragmentation with a dynamic exclusion for 40 s with a 10 ppm tolerance. Data-dependent acquisition parameters were set as resolution power of 17.5 K, isolation window of 2.0 Th, normalized collision energy (NCE) of 27, the maximum injection time of 150 ms, AGC target of 1 × 10^5^, and fixed first mass of m/z 110. For targeted proteomics, an inclusion list containing all the precursor ions of peptide of interest was constructed. Each sample was acquired in technical triplicates.

### Data Analysis

In discovery proteomics analysis, protein identification was performed using MaxQuant (1.5.6.5) against SwissProt human database with 1% false discovery rate (FDR) at protein level. The first search peptide tolerance for precursor and product ion were 20 ppm and 0.02 Da, respectively. The maximum missed cleavages per peptide was 2. Fixed modification was set as carbamidomethylation of cysteine residues (+57.0215 Da). Oxidation of methionine (+15.9949 Da) was selected as variable modifications. The intensity obtained from label-free quantification in MaxQuant was uploaded into Perseus for advanced downstream analysis. Firstly, the reverse protein sequences created during database search, the most common contaminants in the MS analyses, and proteins identified only by modified sequences were removed. Secondly, proteins identified based on only one peptide and proteins for which no unique peptide was identified were removed. To ensure accurate quantification, proteins identified in each sample along three technical replicates were normalized to the summed intensity, followed by determination of the ratio between preclinical AD and healthy control as well as statistical analysis of Student’s *t*-test. Gene ontology (GO) analysis of the significantly regulated proteins in preclinical AD patients was performed by DAVID, which is a tool to provide enrichment analysis of the most relevant GO terms associated with a given list (Huang et al., 2009).

In targeted proteomics analysis, protein identification was performed on Peaks Studio 7 software (Bioinformatics Solutions, Inc., Waterloo, ON, Canada). The data refinement was applied to correct precursor mass by default. All the raw files were searched against UniProt *Homo sapiens* reviewed database with trypsin as digestion enzyme. The error tolerance for precursor mass was 25 ppm using monoisotopic mass and 0.02 Da for fragment ion. The maximum missed cleavages per peptide was two, allowed to be cleaved at both ends of the peptides. Fixed modification was set as carbamidomethylation of cysteine residues (+57.0215 Da). iDiLeu labels (+141.1154 Da for d0, +144.1313 Da for d3, +147.1409 Da for d6, +150.1631 Da for d9, and +153.1644 Da for d12) of peptide N-termini and lysine residues, as well as oxidation of methionine (+15.9949 Da) were selected as variable modifications. Estimation of FDR was enabled. Peptides with FDR < 1% were considered to be unambiguous identification. Peak areas generated by Genesis peak detection algorithm in Thermo Xcalibur 2.2 software were used for quantification. The precursor ion integration tolerance was 15 ppm. Retention time of extracted ion chromatogram of 5-plex iDiLeu-labeled peptides was required to be within 2 min. Isotopic interference correction factors were applied to each sample to rectify the raw values.

## Results and Discussion

### Label-Free Quantification for Discovery Proteomics

**FIGURE 2:**
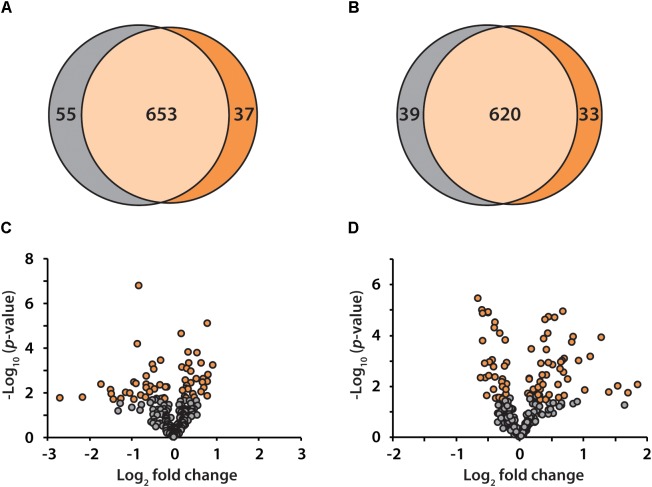
Venn diagram illustration of proteins identified in healthy controls (gray) and preclinical AD patients (orange) for women **(A)** and men **(B)**, respectively. Volcano plots of quantified proteins in women **(C)** and men **(D)**.

To discover candidate protein biomarkers for early diagnosis of AD in preclinical stage, label-free based discovery proteomics displayed in Figure 1 was employed for measurement of protein changes between healthy and preclinical AD individuals as a faster and cleaner technique. Given sex differences in the risk of AD (Mielke et al., 2014), the study was designed so that gender could be taken into account in all the analyses. As shown in Figure 2A, 708 and 690 proteins were identified in healthy and preclinical AD for women with 1% protein-level FDR, respectively. The identified proteins in men were comparable to women (659 and 653 proteins in each group, Figure 2B). Taken collectively, 732 and 704 proteins with more than one unique peptide were identified in healthy controls and preclinical AD patients.

**FIGURE 3:**
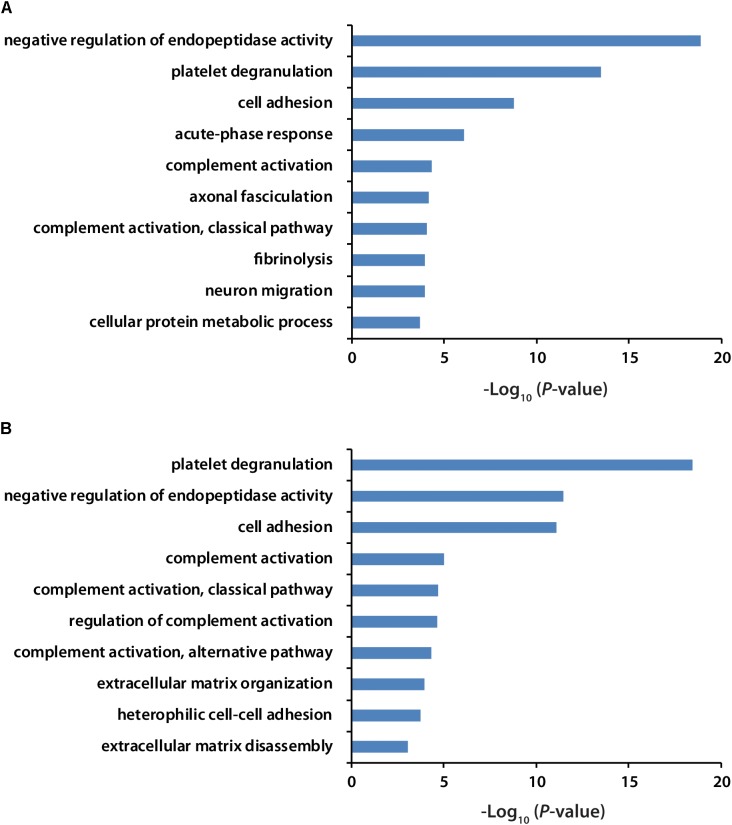
GO analysis of significantly regulated proteins in women **(A)** and men **(B)** with preclinical AD. GO term with Fisher Exact *p*-value smaller than 0.05 was considered to be a strongly enriched biological process.

All the identified proteins were submitted to Perseus for advanced downstream analysis (Tyanova et al., 2016). To ensure accurate quantification, proteins identified based on only one peptide were removed. Proteins identified in sex-matched three pairs of healthy and preclinical AD subjects along with all the technical replicates were utilized for label-free quantification. As shown in volcano plots (Figures 2C,D), 79 and 98 proteins were significantly altered in preclinical AD for women and men in comparison to corresponding healthy controls, respectively (Supplementary Tables S1A,B). They were sequentially used for gene ontology (GO) enrichment analysis to understand what biological processes were statistically over-represented. The top 10 biological processes with Fisher Exact *p*-value < 0.05 in preclinical AD for women and men were shown in Figure 3. Negative regulation of endopeptidase activity, platelet degranulation, and cell adhesion were the three most prominent biological processes in both women and men, all of which were related to AD pathology. Many endopeptidases are capable of proteolyzing Aβ, especially neprilysin, which exhibits the most potent Aβ-degrading activity (Shirotani et al., 2001). Down-regulated neprilysin in the hippocampus and cerebral cortex has been reported in AD development (Shirotani et al., 2001). Platelet degranulation played an important role in platelet-mediated amyloid beta (Aβ) oligomerization (Donner et al., 2016). It was suggested that Aβ stimulated aberrant neuritic growth by activation of cell adhesion signaling pathways (Cotman et al., 1998; Grace et al., 2002).

Many of the differentially expressed proteins have been demonstrated to have similar up- or down-regulation patterns in earlier studies of MCI or AD dementia, including elevated amyloid beta precursor protein in women with preclinical AD (*p*-value = 0.006), which could be further proteolytically cleaved into neurotoxic Aβ42 peptide (Zlokovic, 2005; Sleegers et al., 2006). Secretogranin II, a precursor for neuropeptide secretoneurin, widely distributed in endocrine and nervous tissues, was decreased (*p*-value = 0.00003) in men with preclinical AD as compared to healthy subjects (Li et al., 2008). Interestingly, some proteins displayed opposite regulation trends between women and men in preclinical AD. Neurosecretory protein VGF (VGF), synthesized by neurons where it promotes dendritic growth and survival of cortical neurons (Sato et al., 2012), was significantly down-regulated in men with preclinical AD but increased in women. Apolipoprotein E (apoE) has three common isoforms, all of which differentially modulate Aβ aggregation and clearance (Bu, 2009). ApoE was up-regulated in women with preclinical AD patients but slightly decreased in men. Biomarker candidates of VGF and apoE determined from discovery proteomics need to be further validated through isotopic labeling-based targeted proteomics.

### Quantitative Performance of 5-Plex iDiLeu Labeling Strategy

To demonstrate the strong performance of 5-plex iDiLeu labeling strategy, a mixture of 5-plex iDiLeu-labeled peptide standards were employed for characterization and quantification. The starting material, L-leucine or isotopic L-leucine (L-leucine-1-^13^C, ^15^N) used for iDiLeu synthesis are commercially available, but the slight impurities of these reagents can diminish quantification accuracy. Furthermore, when 5-plex iDiLeu reagents only label the N-terminus of a peptide with +2 charge state, the m/z of five monoisotopic precursors will be 1.5 Th apart, which will inevitably introduce isotopic interference. Therefore, correction factors need to be firstly determined for each of the 5-plex iDiLeu tags to ensure accurate quantification (Supplementary Table S2). A series of equations based on i-Tracker were set up to rectify raw values with correction factors (Shadforth et al., 2005; Greer et al., 2015). Each channel has its own correction equation, where the peak area of both monoisotopic precursor and any possible interferences from neighboring isotopic precursor were taken into consideration.

Peptide standards were labeled with 5-plex iDiLeu reagents. The combination of 5-plex iDiLeu labeled peptides was subjected to LC–MS/MS analysis in triplicates at the ratio of 1:1:1:1:1. Extracted ion chromatograms of precursor ions were used for quantification of 5-plex iDiLeu-labeled peptides. The median ratio of d0:d3:d6:d9:d12 was 0.89:1.08:0.87:0.85:1.00 as shown in the boxplots (Supplementary Figure S2). Medians displayed high accuracy with <15% error, which confirms quantification based on MS^1^-precursor level was reliable in high-resolution and accurate mass (HRAM) measurement by Q-Exactive Orbitrap mass spectrometer.

**FIGURE 4:**
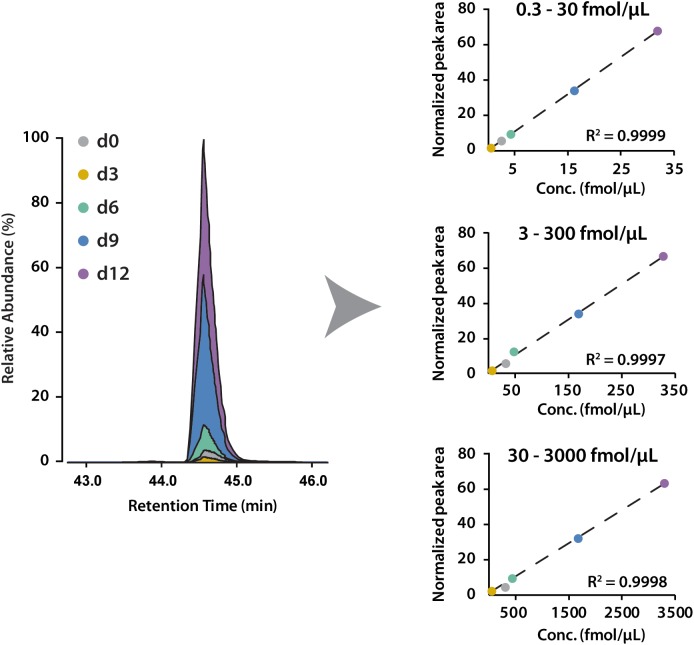
Calibration curves of 5-plex iDiLeu labeled VGF peptide in triplicates. The normalized peak area of precursor ions plotted as a function of peptide concentrations at 0.3–30, 3–300, 30–3000 fmol/μL.

To determine the linear concentration range of this iDiLeu labeling strategy, standards of VGF peptide and tryptic apoE peptides were individually labeled with 5-plex iDiLeu tags and combined at the ratio of 1:10:50:100. The highest concentration stock of VGF peptide ranged from 30 to 3000 fmol/μL. This stock solution was serially diluted twice to prepare concentrations of 3–300 and 0.3–30 fmol/μL. Figure 4 displayed peak area of iDiLeu-labeled VGF peptide at the MS^1^ level as a function of concentration across LC–MS/MS analysis in triplicates by normalization to d9 channel. Each calibration curve revealed a linear response comprising R^2^ around 0.999 spanning two orders of magnitude. The higher the concentration, the lower the coefficient of variation (CV). The CV was below 0.8% in the concentration of 30–3000 fmol/μL but increased to be 8% among the triplicates in 0.3–30 fmol/μL. The limits of quantification for VGF peptide and tryptic apoE peptides were all around 0.1 fmol/μL. The high linearity and low limit of quantification confirmed that iDiLeu tagging is a robust strategy for absolute quantification.

### Absolute Quantification of Biomarkers in Preclinical Alzheimer’s Disease

The iDiLeu labeling strategy has been evaluated with high accuracy and linearity, allowing for verification of biomarker candidates in CSF samples. We set up the targeted proteomics strategy to perform quantification in both healthy and preclinical AD subjects using 5-plex iDiLeu reagents (Figure 1). Each CSF protein digests was labeled with d0 channel, respectively. Peptide standards were composed of peptides derived from proteins VGF and apoE. The standards were labeled with d3, d6, d9, d12, individually, followed by combination at the ratio of 1:10:50:100. The four channels labeled peptide standards were spiked into each CSF sample with equal amount at the same ratio. The final concentration of peptide standard was ranged from 0.04 to 4 pmol/μL for proteins VGF and apoE.

**FIGURE 5:**
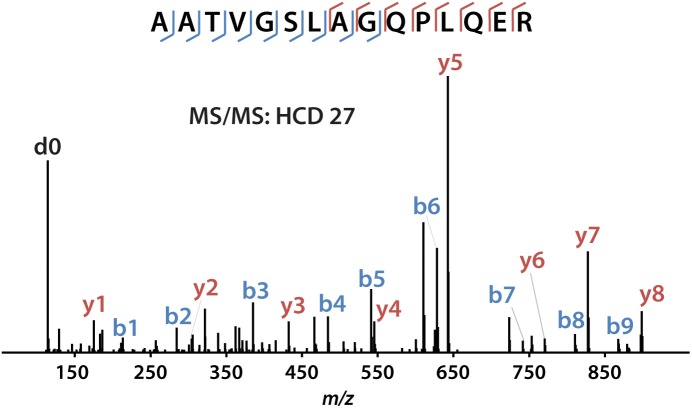
Example of tandem mass spectrum for iDiLeu d0-labeled apoE peptide in CSF.

**FIGURE 6:**
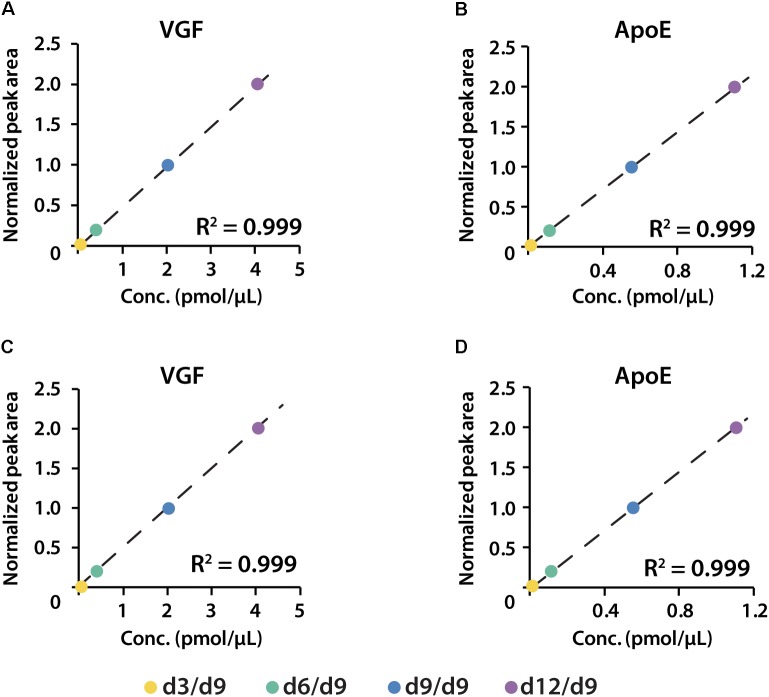
Calibration curves of proteins VGF and apoE constructed in both healthy **(A,B)** and preclinical AD **(C,D)** subjects.

iDiLeu labeled peptides were fragmented under NCE of 27. Figure 5 displayed representative tandem mass spectrum of d0-labeled peptide AATVGSLAGQPLQER in CSF sample. iDiLeu d0 tag was fragmented to produce dimethylated immonium reporter ion at *m/z* of 114.1. Abundant b- and y- product ions have been matched to specific peptide sequence within an FDR < 1%, which is equivalent to a -10lgP score of 15. This score is derived from the *p*-value that indicates the statistical significance of the peptide-spectrum match (Zhang et al., 2012). Peptide AATVGSLAGQPLQER of apoE had -10lgP score of 58.13, which presented as a high-quality spectrum with modification of iDiLeu tag. The plot of normalized peak area of each peptide against concentration displayed high linearity in both preclinical AD and control samples, which ensured the accuracy of biomarker quantification (Figure 6). A standard deviation within 3% was demonstrated across the triplicates run in each sample.

**FIGURE 7:**
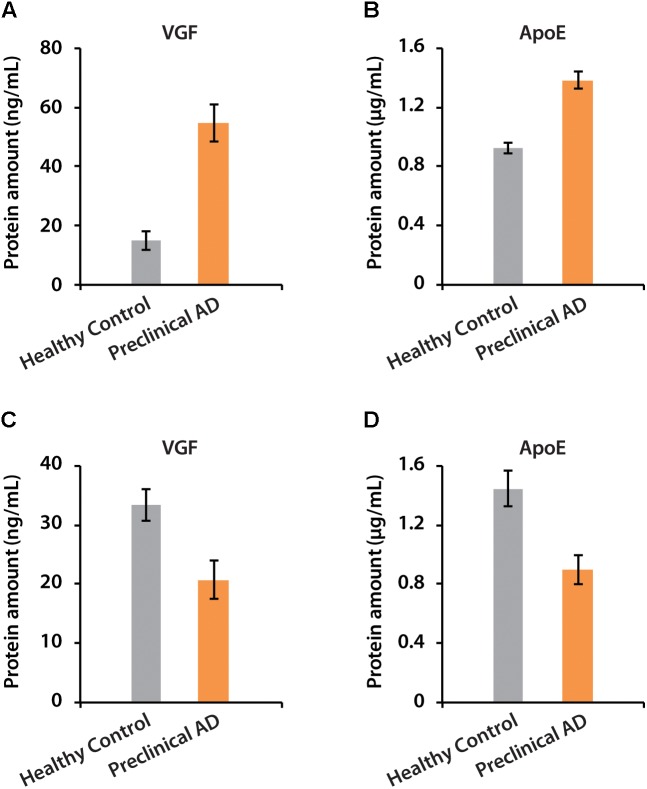
The determination of absolute amounts of proteins VGF and apoE in CSF of women **(A,B)** and men **(C,D)**.

The amounts of VGF were quantified as 55 and 21 ng/mL in CSF of women and men with preclinical AD, respectively. ApoE has higher abundance than VGF, which were 1.38 and 0.9 μg/mL in preclinical AD for women and men, respectively (Figure 7). The isotopic labeling based targeted proteomics suggested that both VGF and apoE were down-regulated in men with preclinical AD but showing opposite regulation pattern in women, which is consistent with that of label-free based discovery proteomics. The average ages for women were younger than men, which may partially contribute to the opposite phenomenon as aging is the greatest risk factor for AD. Moreover, women had significantly higher glucose metabolism in the brain areas involved in the pathological process of AD, while men showed consistent level of cognitive impairment (Perneczky et al., 2007). Sex difference in cortical cytoarchitecture, such as brain size, neuron account, and neuron density, may also lead to brain cognitive reserve difference in women and men (Rabinowicz et al., 2002).

There is no clear consensus regarding whether VGF and apoE are positively or negatively associated with AD dementia (Hesse et al., 2000; Carrette et al., 2003; Liu et al., 2013; Busse et al., 2015; Hölttä et al., 2015). Statistical analysis demonstrated that VGF was significantly down-regulated in men with preclinical AD, possibly due to its regulation of hippocampal function and memory, in part through modulation of brain-derived neurotrophic factor (BDNF) secretion and signaling (Bozdagi et al., 2008). The increased expression of VGF in women may be correlated to immune manifestations with negative effects on brain function and neuronal repair processes, as reported that VGF was expressed with a higher percentage of peripheral CD3+ T cells from AD patients compared to age-matched healthy controls (Akiyama et al., 2000; Busse et al., 2015).

ApoE is a lipoprotein containing 299 amino acids, regulates lipid homeostasis by mediating lipid transport between various cells in the brain. The delivering function of Aβ peptide is thought to initiate toxic events, leading to synaptic dysfunction and neurodegeneration in AD (Liu et al., 2013). Analysis of apoE alleles in AD demonstrated that there was a significant association of 𝜀4 allele with an earlier age of AD onset, resulting from domain interactions in the presence of arginine at residue 112 (Kim et al., 2009; Wolf et al., 2013). ApoE may function as Aβ chaperones for receptor-mediated clearance of soluble extracellular Aβ but may also lead to intraneuronal Aβ accumulation (Lane and Farlow, 2005). The absolute amount and particular isoform of apoE may influence both Aβ fibrillogenesis and metabolism to differing extents. It has been reported that increased cytokines and decreased cholesterol in the brain could reduce apoE levels in AD (Gueguen et al., 2001). However, higher levels of apoE have also been reported in CSF of AD patients compared with age-matched controls (Lindh et al., 1997; Sihlbom et al., 2008). According to our results, 1.38 and 0.9 μg/mL of apoE could be defined as thresholds for women and men with preclinical AD, respectively, but a large cohort study would be necessary to determine the threshold of multiple biomarkers to aid pre-clinical diagnosis.

## Conclusion

In summary, we reported a large number of proteins identified in human CSF. A panel of potential protein biomarkers was produced to differentiate healthy controls and preclinical AD patients in this pilot study. Many of the candidate biomarkers have shown similar trends in patients with MCI or AD, including amyloid precursor protein, apolipoprotein E, and several neuroendocrine proteins. More interestingly, the differential expression of proteins in women and men with preclinical AD might provide us with a novel perspective to reveal the pathological mechanism of AD, but a larger cohort study is necessary to further elucidate the correlation between AD and gender differences. For the first time, the novel iDiLeu-enabled standard curve approach has been established for absolute quantification of candidate protein biomarkers in each CSF sample with high accuracy and reproducibility. Taken together, we established a comprehensive strategy to discover and verify candidate biomarkers by integrating label-free based discovery proteomics with isotopic labeling based targeted proteomics. This strategy could be applied for biomarker discovery and verification across a broad spectrum of diseases.

## Author Contributions

XZ, JW, and LL designed the research. XZ and JW performed the experiments and analyzed the data. HZ, CC, and OO collected and provided the CSF specimens. XZ, HZ, and LL prepared the manuscript and all authors provided feedback.

## Conflict of Interest Statement

The authors declare that the research was conducted in the absence of any commercial or financial relationships that could be construed as a potential conflict of interest.
