# Supplementary material for: A Strategy for Discovery and Verification of Candidate Biomarkers in Cerebrospinal Fluid of Preclinical Alzheimer’s Disease
Source: Front Mol Neurosci. 2019 Jan 7;11:483. doi: 10.3389/fnmol.2018.00483 (PMC6330998; doi:10.3389/fnmol.2018.00483)
Supplement: Supplementary file 1 [file Data_Sheet_1.docx]

**Discovery and verification of candidate biomarkers in cerebrospinal fluid of preclinical Alzheimer’s disease**

Xiaofang Zhong^1†^, Jingxin Wang^2†^, Cynthia Carlsson^3^, Ozioma Okonkwo^3^, Henrik Zetterberg^4,5,6,7^, Lingjun Li^1,8*^

^1^School of Pharmacy, University of Wisconsin-Madison, Madison, Wisconsin, USA.

^2^Neuroscience Training Program, University of Wisconsin-Madison, Madison, Wisconsin, USA.

^3^School of Medicine and Public Health, University of Wisconsin, Madison, Wisconsin, USA.

^4^Institute of Neuroscience and Physiology, Sahlgrenska Academy, University of Gothenburg, Gothenburg, Sweden.

^5^Clinical Neurochemistry Laboratory, Sahlgrenska University Hospital, Mölndal, Sweden.

^6^Department of Neurodegenerative Disease, UCL Institute of Neurology, London, UK.

^7^UK Dementia Research Institute at UCL, London, UK

^8^Department of Chemistry, University of Wisconsin, Madison, Wisconsin, USA.

^†^These authors contributed equally to this work.

*Correspondence should be addressed to L.L. (lingjun.li@wisc.edu)

**Supplementary Materials**

**Supplementary Figure S1.** Structure of isotopic *N,N*-dimethyl leucine (iDiLeu) tags.

**Supplementary Figure S2.** Boxplots of log2 fold change of 5-plex iDiLeu labeled peptide standards at ratio of 1:1:1:1:1. The median ratio from precursor quantification was 0.89:1.08:0.87:0.85:1.00. The lower and upper inner fence of the box represent the first and third quartile. The whiskers expand to the most extreme data point with a coefficient value of 1.5x interquartile range.

**Supplementary Table S1.** Significantly regulated proteins in female (A) and male (B) preclinical AD patients compared to corresponding healthy controls.

**Supplementary Table S2.** Primary and isotopic peak fractions for iDiLeu-labeled target peptides.

**Supplementary Figures and Tables**

**
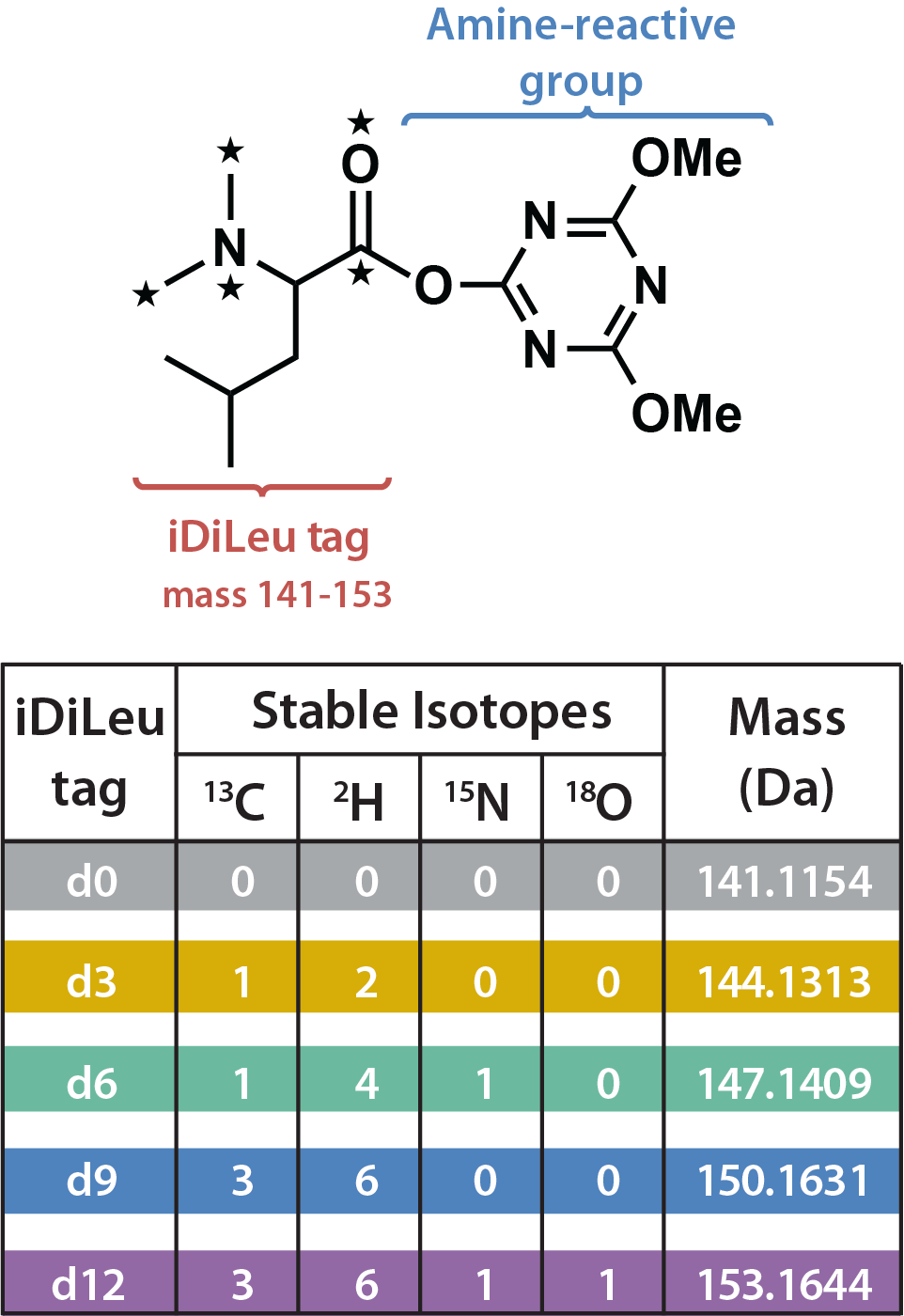
**

**Figure S1.**


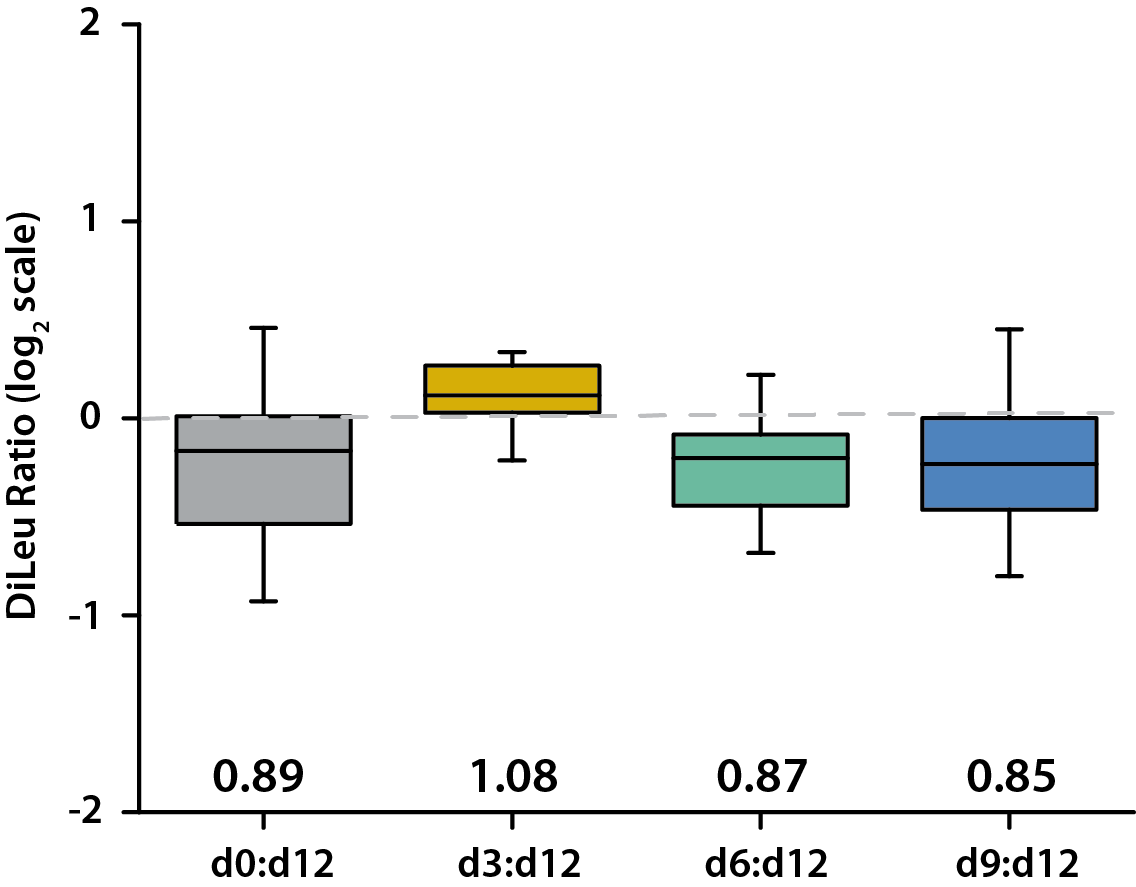


**Figure S2.**

**Table S2.** Primary and isotopic peak fractions for iDiLeu-labeled target peptides.


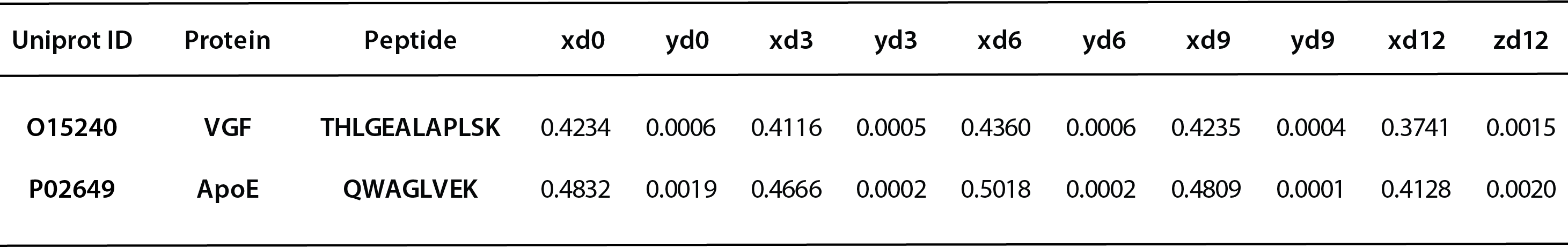


x, y, and z represent the percentages of monoisotopic peak, interference peak to the heavier mass-labeled peptide, and interference peak to the lighter mass-labeled peptide, respectively.
